# Usage of Adenovirus Expressing Thymidine Kinase Mediated Hepatocellular Damage for Enabling Mouse Liver Repopulation with Allogenic or Xenogenic Hepatocytes

**DOI:** 10.1371/journal.pone.0074948

**Published:** 2013-09-24

**Authors:** Daniel Moreno, Anangi Balasiddaiah, Oscar Lamas, Cedric Duret, Leire Neri, Laura Guembe, Miguel Galarraga, Esther Larrea, Martine Daujat-Chavanieu, Jordi Muntane, Patrick Maurel, Jose Ignacio Riezu, Jesus Prieto, Rafael Aldabe

**Affiliations:** 1 Gene Therapy and Hepatology Area, Center for Applied Medical Research (CIMA), University of Navarra, Pamplona, Spain; 2 Institut National de la Sante et de la recherche Medicale, U1040, Montpellier, France; 3 Université Montpellier 1, UMR-S1040, France; 4 CHU Montpellier, Institut de Recherche en Biotherapie, Hopital Saint Eloi, Montpellier, France; 5 Department of Morphology, Center for Applied Medical Research (CIMA), University of Navarra, Pamplona, Spain; 6 Department of Imaging, Center for Applied Medical Research (CIMA), University of Navarra, Pamplona, Spain; 7 Liver Research Unit, Instituto Maimónides para la Investigación Biomédica de Córdoba (IMIBIC), Reina Sofia University Hospital, Córdoba, Spain; 8 CIBER-EHD, University Clinic, University of Navarra, Pamplona, Spain; 9 Liver Unit, University Clinic, University of Navarra, Pamplona, Spain; Saint Louis University, United States of America

## Abstract

It has been shown that the liver of immunodeficient mice can be efficiently repopulated with human hepatocytes when subjected to chronic hepatocellular damage. Mice with such chimeric livers represent useful reagents for medical and clinical studies. However all previously reported models of humanized livers are difficult to implement as they involve cross-breeding of immunodeficient mice with mice exhibiting genetic alterations causing sustained hepatic injury. In this paper we attempted to create chimeric livers by inducing persistent hepatocellular damage in immunodeficient Rag2^-/-^ γc^-/-^ mice using an adenovirus encoding herpes virus thymidine kinase (AdTk) and two consecutive doses of ganciclovir (GCV). We found that this treatment resulted in hepatocellular damage persisting for at least 10 weeks and enabled efficient engraftment and proliferation within the liver of either human or allogenic hepatocytes. Interestingly, while the nodules generated from the transplanted mouse hepatocytes were well vascularized, the human hepatocytes experienced progressive depolarization and exhibited reduced numbers of murine endothelial cells inside the nodules. In conclusion, AdTk/GCV-induced liver damage licenses the liver of immunodeficient mice for allogenic and xenogenic hepatocyte repopulation. This approach represents a simple alternative strategy for chimeric liver generation using immunodeficient mice without additional genetic manipulation of the germ line.

## Introduction

Experiments involving human hepatocytes are essential for the development of new drugs and for unraveling fundamental biological mechanisms underlying metabolic and viral diseases [1]. In vitro cultures of primary cells are not suitable for metabolic or virological studies as human hepatocytes rapidly dedifferentiate losing the mature metabolic phenotype and the ability to support replication of hepatotropic viruses such as HCV and HBV [2,3]. Several studies have documented that transplanted human hepatocytes can integrate into the murine liver parenchyma and that this chimeric organ may serve as a valuable tool for biological, virological and pharmacological studies requiring the use of human hepatic cells [4-6]. During the last years considerable efforts have been made to establish systems for efficient repopulation of the livers of immunodeficient mice with human hepatocytes. However, all of the murine models enabling successful hepatic engraftment of human hepatocytes and effective liver repopulation with the xenogenic cells were based on cross-breeding of immunodeficient mice with mouse strains harboring genetic defects determining persisting hepatocellular damage. Of these, the most well characterized models include those involving the uPA transgene [7-9] or FAH deficiency [10]. With either of the two models, several studies have reported that the transplanted hepatocytes were able to replace about 70% of the host parenchyma and in some cases up to 90% [11] resulting in the presence of substantial amounts of human albumin in the serum of these animals and rendering the sera protein profiles of chimeric mice more human-like [12]. Furthermore, these mice also exhibited a humanized profile of drug metabolism and excretion, thus representing useful tools for pharmacological studies [13-16]. Moreover the possibility to infect the humanized livers with HBV or HCV contributed to enrich the knowledge of the host-pathogen interactions and helped the development and characterization of new antiviral drugs [17-21].

Despite the practical advantages of generating humanized mouse livers, there are few data on the organization of human hepatocytes within the regenerative nodules, the interaction of mouse sinusoidal endothelial cells with human hepatocytes and the architecture of the regenerative nodules. Data from different studies indicate that the transplanted cells are unable to interact appropriately with the host environment. This is suggested by the presence of the glycogen accumulation in human hepatocytes [22], the development of hepatic steatosis after prolonged times following transplantation [23] and by the altered pattern of hepatocyte growth [24]. Also it has been reported that there is a sparse number of sinusoidal cells inside the regenerating nodules of human hepatocytes [25,26].

It has been shown that transgenic animals expressing hepatocyte-targeted HSV-Thymidine kinase (HSV-Tk) experienced hepatocellular damage upon GCV administration allowing engraftment and liver repopulation of foreign hepatocytes [27,28]. Here we show that the inoculation to immunodeficient mice of an adenovirus encoding HSV-Tk followed by GCV administration was sufficient to generate sustained hepatocellular damage and to create the conditions enabling engraftment of allogenic or xenogenic hepatocytes and generation of chimeric livers. This simple method circumvents the complexities of producing mice strains combining the immunodeficient status and a genetic modification causing persistent liver damage and opens the possibility to generate chimeric livers in animal species that are not genetically modified. Additionally we also show that the functionality of the humanized liver is limited to a period of time of around 12 weeks since, in the long term, continuous growth of xenogenic hepatocytes is hampered by reduced integration of murine CD31^+^ cells into the regenerative human nodules and also by disturbed polarization of the human cells.

## Materials and Methods

### Adenovirus preparation

The recombinant adenoviral vector (AdTk) encoding the herpes simplex virus thymidine kinase (HSV-Tk) (provided by Dr G. Gonzalez-Aseguinolaza, CIMA) was expanded in 293 cells and purified by cesium chloride ultracentrifugation. The purified virus was passed through PD-10 columns to remove residual salts, and the viral particles were eluted in Tris-HCL. The viral aliquots were stored at -80°C. The viral titer was determined using real-time PCR and a plaque assay.

### The induction of liver damage

Male Balb/C Rag2^tm1Fwa^ Cd3g^tm1Amk^ (Rag2^-/-^ γc^-/-^) (5-week old) mice were intravenously inoculated with 5 X 10^9^ PFU of recombinant AdTk via the retro-orbital plexus in 100 µl of saline solution. Three days after infection, the mice were administrated 2 doses of 25 mg/kg of GCV (Roche) intraperitoneally on alternating days.

### The isolation and transplantation of mouse and human hepatocytes

Murine hepatocytes were obtained from 7- to 9-week-old transgenic C57BL/6 Tg14 (act-eGFP) Os-bY01 mice using the two-step collagenase perfusion technique. The human hepatocytes were isolated as previously described also using the two-step collagenase procedure [29] either from liver resections obtained from patients submitted to surgical intervention for primary or secondary liver tumor resection under the approval of the "Ministère de l’Enseignement Supérieur et de la Recherche" (reference: MESR DC-2008-531) and the “Comisión de Ética e Investigación Sanitaria del Hospital Universitario "Reina Sofía", with the patient’s written consent and the French and Spanish authorities. Finally, the hepatocytes were frozen or plated in collagen type 1-coated flasks and sent to Pamplona by mail. Plated human hepatocytes were maintained in culture between 5 and 10 days after isolation before being transplanted. All the used hepatocyte preparations presented a viability superior to 75%.

All of the animal procedures were performed in accordance with Institutional guidelines and were approved by the Animal Experimentation Ethical Committee of the University of Navarra (Permit number: 041-07 and 095-09). Forty-eight hours after the administration of a second dose of GCV, the male Balb/C Rag2^tm1Fwa^ Cd3g^tm1Amk^ mice were anesthetized with isoflurane (Abbott labs) and kept on a heating pad during the entire procedure. The mice were injected with ketofen (Merial labs) intramuscularly at 5 mg/kg to decrease pain and inflammation. Next, a mid-abdominal incision was made, and 1-2 X 10^6^ murine or human viable hepatocytes resuspended in 100 µl of PBS were intrasplenically transplanted, followed by the closure of the abdominal incision using silk sutures. Next animals transplanted with human hepatocytes were intravenously administered 100 µl of human AB serum (SIGMA) for improving hepatocyte engraftment, as in a preliminary experiment we observed a small enhancement in human hepatocyte engraftment. The transplanted mice were then treated with enrofloxacin (Bayer Health Care) administered at 25 mg/ml in drinking water for one week to protect them from pathogenic microorganisms. All efforts were made to minimize suffering.

### Serum biochemical analysis

The physiological functions of the host parenchymal cells and the transplanted human hepatocytes were examined by the assessment of the serum transaminase and human albumin levels, respectively. The peripheral blood samples were collected via retro-orbital bleeding once per week, and the serum was separated from whole blood after centrifugation at 8000 rpm for 15 minutes. The serum alanine and aspartate aminotransaminase (ALT, AST) levels were quantified using a HITACHI C311 analyzer. The serum human albumin levels were determined using the Human Albumin ELISA quantitation kit (Bethyl laboratories).

### Histological analysis

The livers were harvested at the indicated time points and cut into 3 mm portions, some of which were formalin-fixed, paraffin-embedded and cut in 3-µm thick sections while others were immediately frozen in OCT (Sakura). Paraffin sections were stained with hematoxylin and eosin (HE) for routine histology and picrosirius red to evaluate liver fibrosis. In addition, immunohistochemistry was applied using the following primary antibodies: rat anti-mouse CD45 (leukocytes) (1:500) (BioLegend), rat anti-mouse F4/80 (macrophages) (1:400) (eBiosciences), rat anti-mouse NIMP-R14 (neutrophils) (1:10000) (Abcam), mouse anti-smooth muscle actin (SMA) (activated stellate cells) (1:40000) (Sigma), rat anti-mouse A6 (oval cells) (1:50) (kindly provided by Dr V. Factor, National Institute of Health), rat anti-mouse CD10 (1:400) (Santa Cruz Biotechnology), rat anti-mouse CD31 (endothelial cells) (1:50) (Dianova), rabbit anti-TK (1:8000) (provided by Dr WC Summers, Yale University), mouse anti-human mitochondria (MTCO2) (1:2000) (Abcam), mouse anti-human cytokeratin 18 (hCK18) (Dako, Golstrup, Denmark), goat anti-human albumin (hAlb) (1:8000) (Bethyl Labs) and rabbit anti-green fluorescent protein (GFP) (1:4000) (Abcam). In some cases, antigen retrieval was applied: 2 µg/ml proteinase K (Sigma), 37 °C, 30 min (for F4/80 and NIMP-R14) or heating for 30 min at 95 °C in 0,01 M Tris-1 mM EDTA pH 9 in a Pascal pressure chamber (Dako) (for SMA, CD10, CD31, TK, MTCO2, hCK18 and GFP). In the case of rat and goat primary antibodies, sections were first incubated with rabbit anti-rat (Dako) or rabbit anti-goat (Dako) secondary antibodies. Then, the EnVision system (Dako) was used in all cases according to manufacturer instructions. For the visualization of lipid accumulation in the hepatocytes, 10-µm thick frozen sections were cut, fixed in formalin and stained with fat red (Sigma). In the case of human hepatocytes transplants, consecutive frozen sections of these livers were immunostained with anti-hCK18 antibody as described.

### Image analysis

A set of FIJI V1.46b plugins (a distribution of ImageJ) was developed by the Imaging Core Facility (CIMA, University of Navarra) to analyze the images. These plugins contain the image-processing operations, including color segmentation, filtering and particle counting that can facilitate the segmentation of tissue from the background and can determine the ratio of positive vs. tissue area. Using these FIJI plugins, the collagen, neutrophils, macrophages, leukocytes, endothelial cells, oval cells, stellate cells, polarized hepatocytes, murine hepatocytes expressing GFP, and human hepatocytes present in the murine livers were quantified. The quantification was expressed as the ratio of positive vs. tissue area or as the percentage of the donor parenchyma in the host liver in the case of the quantification of the donor hepatocytes.

### The detection of HCV and HBV

Five to nine weeks after the transplantation of the human hepatocytes, the mice were infected with 100 µl of human sera containing either HCV (1,35 x 10^7^ copies/ml) or HBV (5,7 x 10^7^ copies/ml), bled weekly and analyzed for the presence of HCV and HBV viral particles. Viral nucleic acids were isolated from mice sera with high pure viral nucleic acid kit (Roche Molecular Biochemicals) according to the manufacturer instructions. In case of HCV detection, for reverse transcription, the RNA extracted was first denatured for 1 min at 90 °C. cDNA synthesis was then performed for 1 h at 37 °C in a reaction mixture containing 60 U Moloney murine leukaemia virus reverse transcriptase (MMLV RT; Gibco-BRL), RT buffer, 5 mM dithiothreitol, 2 mM dNTP mix (Roche Molecular Biochemicals), 100 ng random primers (Roche Molecular Biochemicals) and 12 U RNase inhibitor (Promega) in a final volume of 10 µl. Mouse sera were analyzed to detect the genomic HCV RNA by nested PCR from the 5´ non-coding region (5´ NCR). The outer primers used were JL1 (sense: 5´- CTGTGAGGAACTACTGTCT-3´) and JL2 (antisense: 5´ TATCAGGCAGTACCACAAG-3´). The inner primers were JL3 (sense: 5´-ACTGTCTTCACGCAGAAAGC-3´) and JL4 (antisense: 5´-GACCCAACACTACTCGGCTA-3´). The conditions for both rounds of PCR consisted of an initial denaturation at 95°C for 1 minute, followed by 35 cycles of 95°C for 15 s, 52°C for 12 s and 72°C for 20 s, and a final extension at 72°C for 1 min. The reactions were performed using 3 µl of cDNA or of first-round PCR product, inner or outer primers in a final volume of 20 µl. The size of the final PCR product was 200 bp.

For the detection of HBV, DNA was extracted from 100 µl of mouse serum using the High Pure Viral Nucleic Acid Kit (Boehringer Mannheim, Mannheim, Germany). Nested PCR was performed using the following primers: HBV-S1 (sense: 5’-AGAATCCTCACAATACCGCA), HBV-S2 (antisense: 5’-CCCCAATACCACATCATCCA), HBV-S3 (sense: 5’-TCCAATCACTCACCACATCATCCA), and HBV-S4 (antisense: 5’-CCCTACGAACCACTGAACAA). Briefly, the first round of PCR was performed in a total reaction volume of 40 µl containing 25 µl of reaction mix with HBV-S1 and HBV-S2 primers and 15 µl of c-DNA. The second round of PCR was performed in a total reaction volume of 22 µl containing 12 µl of reaction mix with HBV-S3 and HBV-S4 and 10 µl of first-round PCR product. The conditions applied in both PCRs were 95°C for 20 seconds, 36 cycles of 95°C for 20 seconds, 60°C for 15 seconds, and 72°C for 25 seconds, followed by a final extension at 72°C for 10 minutes. The size of the final PCR product was 400 bp.

### Statistical analysis

Statistical analyses were performed using parametric (Student’s t test) tests. All P-values were two-tailed and considered being significant if the associated value was less than 0.05. Descriptive data for continuous variables are reported as means ± standard deviation. GraphPad Prism was used for statistical analysis.

## Results

### The characterization of AdTk/GCV-induced liver damage

We assessed whether liver transduction with AdTk followed by GCV administration could result in a form of chronic liver damage enabling liver repopulation with allogenic or xenogenic hepatocytes.

**Figure 1:**
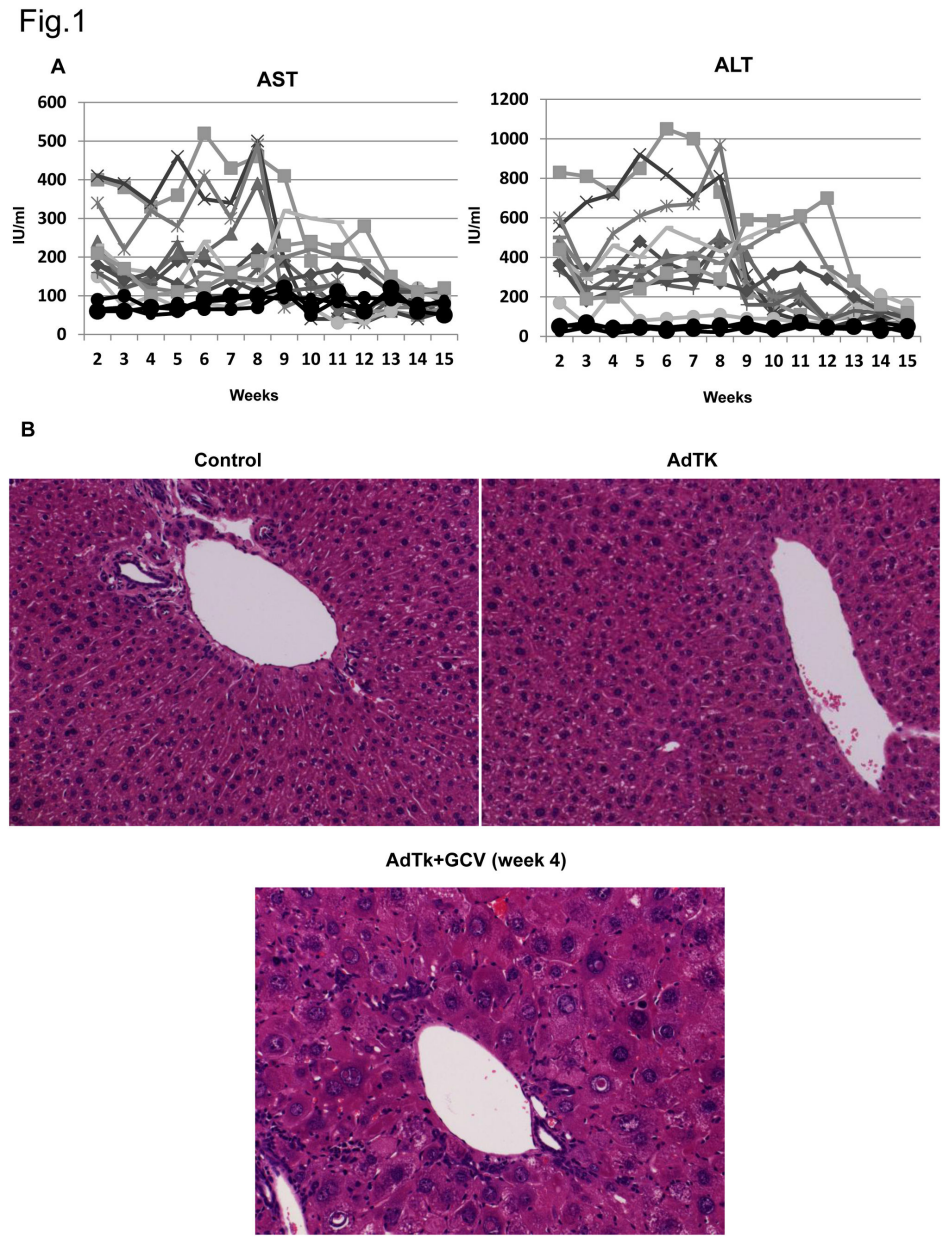
Analysis of liver damage in Rag2^-/-^ IL2γc^-^/^-^ mice treated with AdTk and GCV. A) Animals were bled weekly and aminotransferases (aspartate aminotransferase, AST and alanine aminotransferase, ALT) were measured in 11 AdTk +GCV (25 mg/ml) treated animals represented by independent lines. Control, GCV and AdTk Rag2^-/-^ IL2γc^-^/^-^ animals are represented with black circles and lines, presenting AST values between 50 and 120 IU/ml and ALT values between 20 and 70 IU/ml through all the experiment. Each line represents values in one animal throughout the experimental period. X axis represents the weeks post GCV administration while Y axis represents units per ml. B) Histology of control, AdTk infected and AdTk/GCV treated mice livers at week 4 after GCV administration. Original magnification x200.

**Figure 2:**
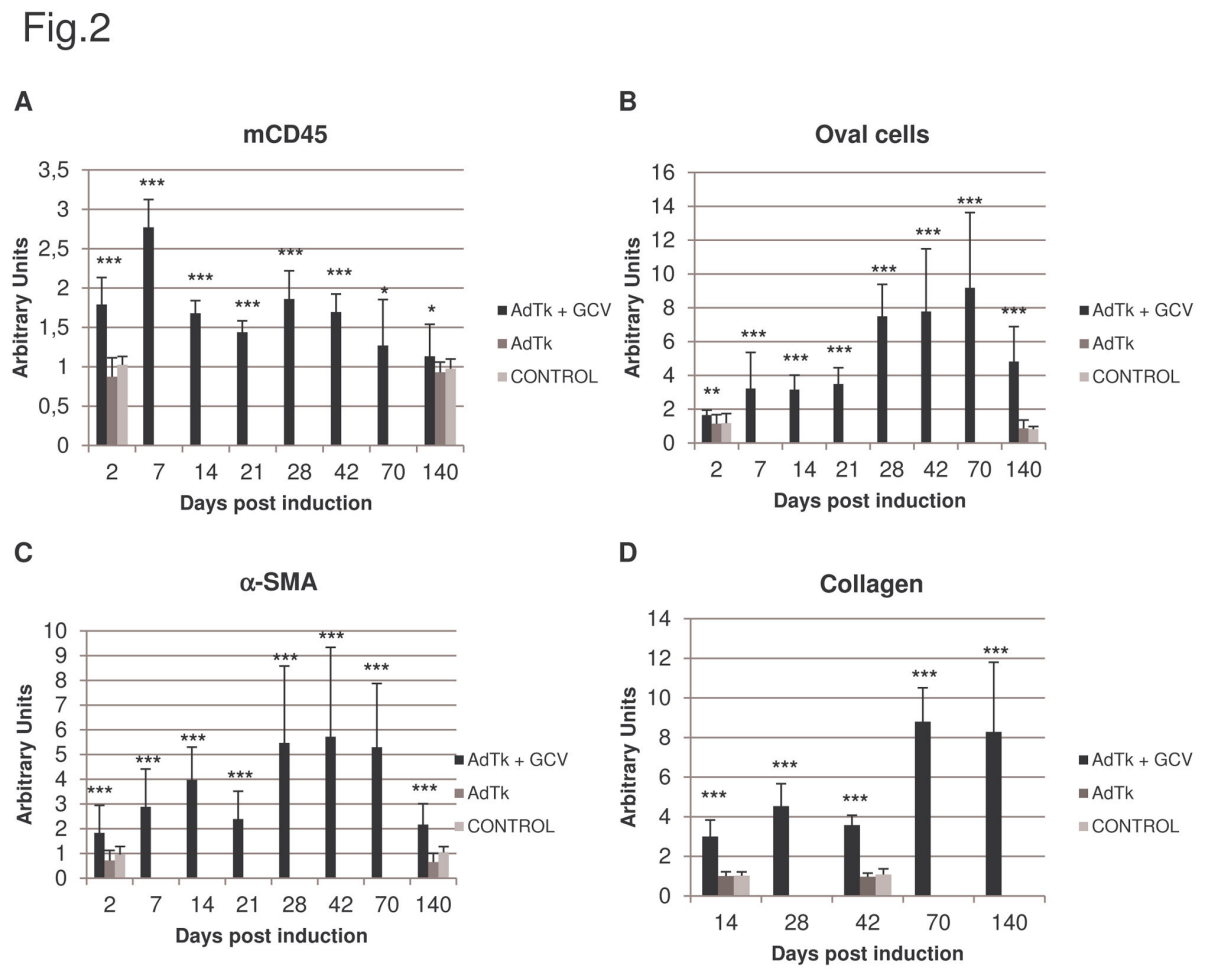
Evaluation of cellular infiltrate present in the liver as consequence of AdTk/GCV mediated liver injury. Control, AdTk alone (AdTk) or treated with GCV (AdTk+GCV) mice were sacrificed at different time points after GCV administration. The livers were collected and immunohistochemistry for leukocytes (CD45 antigen) (A), oval cells (B) and activated stellate cells (C). Sirius Red staining was used for characterizing collagen accumulation (D). Representative examples of immunohistochemically stained sections are shown in Figure S1 and Figure S2 in File S1. Three stained liver sections from 5 animals/time point/group were quantified using FIJI imaging software and obtained results were normalized with control group values. X axis represents the days post GCV administration and liver damage induction while Y axis represents normalized ratio between positive area and total area in arbitrary units. Groups were compared at each time point (* *p*< 0.05, ** *p*< 0.01, *** *p*< 0.001, two-tailed Student’s *t*-test). Results obtained from mice treated with GCV alone are similar to the observed in control and AdTk mice.

We administered 5 X 10^9^ PFU of recombinant AdTk via the retro-orbital plexus to male Rag2^-/-^ γc^-/-^ mice and 3 days later the animals were injected intraperitoneally with 2 doses (25 mg/kg) on alternating days. These AdTk/GCV-treated mice experienced an elevation of ALT and AST levels, not observed in mice treated only with GCV or AdTk, presenting some variability between mice and lasting for 8-9 weeks (Figure 1A) with complete normalization of their values by week 15. Liver histology at different time points showed the presence of swollen hepatocytes with enlarged nuclei only in the livers of AdTk/GCV-treated mice and increased numbers of infiltrating cells dispersed throughout the liver parenchyma that were associated with the induction of liver damage (Figure 1B). The same treatment has been previously reported to cause a similar liver damage in Tk transgenic animals [27]. We identified the cellular infiltration present only in AdTk/GCV-treated mice as leukocytes (CD45^+^ cells), composed mainly of Kupffer cells at early time points and increased number of neutrophils after week 4 (Figure 2A and Figure S1 in File S1).

Interestingly, in the AdTk/GCV-treated Rag2^-/-^ γc^-/-^ mice (Figure 2B), there was a small increase in the number of oval cells, identified as A6 positive cells, during the first 3 weeks after the administration of GCV, which continued to increase from weeks 4 to 10 and decreased from week 10 until the end of the experimental period. Also we observed an increased number of activated stellate cells during the first 6 weeks which progressively decreased thereafter (Figure 2C). In the damaged livers, α-SMA staining was observed in the portal areas but was more prominent intralobularly (Figure S2 in File S1). As shown in Figure 2D, we also observed collagen deposition which was more pronounced at 10 weeks after GCV administration, coinciding with a greater activation HSCs.

### Mouse and human hepatocyte transplantation

**Figure 3:**
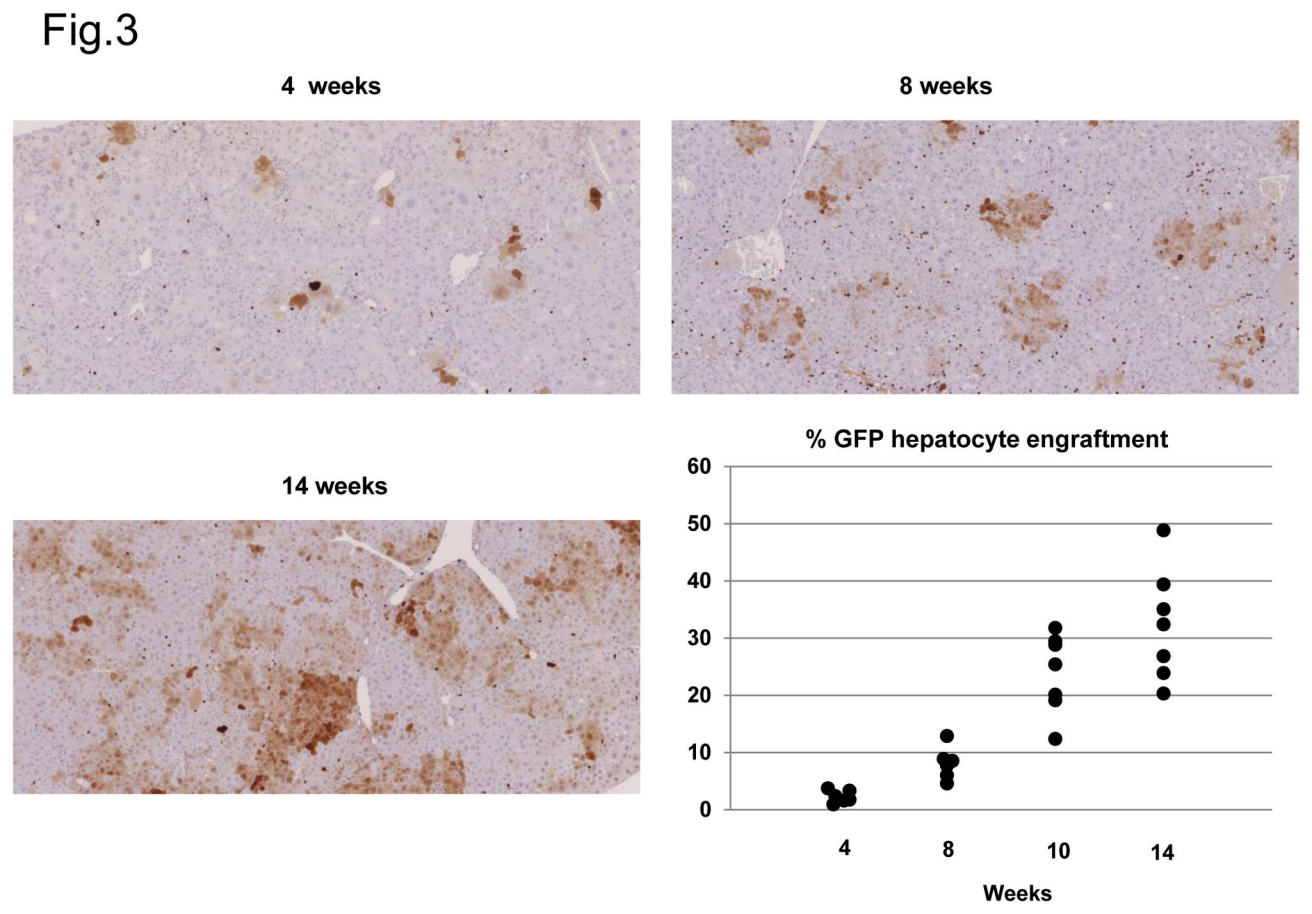
Repopulation of AdTk/GCV treated mice livers with mouse GFP hepatocytes. Mice infected with AdTk and treated with GCV were transplanted with GFP^+^ mouse hepatocytes and 3 animals were sacrificed at each time point after cell transplantation. Thereafter, livers were collected and analyzed for GFP hepatocyte engraftment by immunohistochemical staining. Presented images show GFP expression in hepatic sections from livers collected 4, 8 and 14 weeks after hepatocyte inoculation. GFP positive areas from 2-3 liver sections of each animal were quantified making use of FIJI imaging software and represented as percentage of total section area positive for GFP expression. Original magnification x20.

Several studies have established that chronic hepatocyte elimination can generate the necessary space to license the liver and to allow for the engraftment and expansion of transplanted hepatocytes [5,11]. Consequently, we assessed the potential of AdTk/GCV-induced liver damage to support mouse hepatocyte transplantation, engraftment and replacement. Thus, GFP-positive hepatocytes were transplanted into these mice to identify the donor cells from the host hepatocytes. After transplantation of mouse GFP-positive hepatocytes, the recipient mice were sacrificed at different time points and donor cells were detected by GFP immunostaining. We found that at 4 weeks after transplantation, the GFP hepatocytes were present mainly as individual GFP-positive cells that started forming cell clusters at later time points (Figure 3). As shown in Figure 3, we observed a steady repopulation of the liver by the GFP-positive cells that exhibited a replacement index (RI) between 20% and 50% of the liver parenchyma by week 14 after hepatocyte transplantation. We observed that the engrafted GFP-positive hepatocytes were indistinguishable by its shape and structure from the host hepatocytes being correctly polarized and perfectly integrated into the host parenchyma. There was a homogenous distribution of the liver sinusoids inside the allogenic regenerating nodules and an adequate interaction between grafted cells and sinusoidal endothelial cells (Figure S3 in File S1). Thus, AdTk/GCV-treated livers of immunodeficient mice exhibited the necessary conditions to be efficiently repopulated by allogenic hepatocytes.

**Figure 4:**
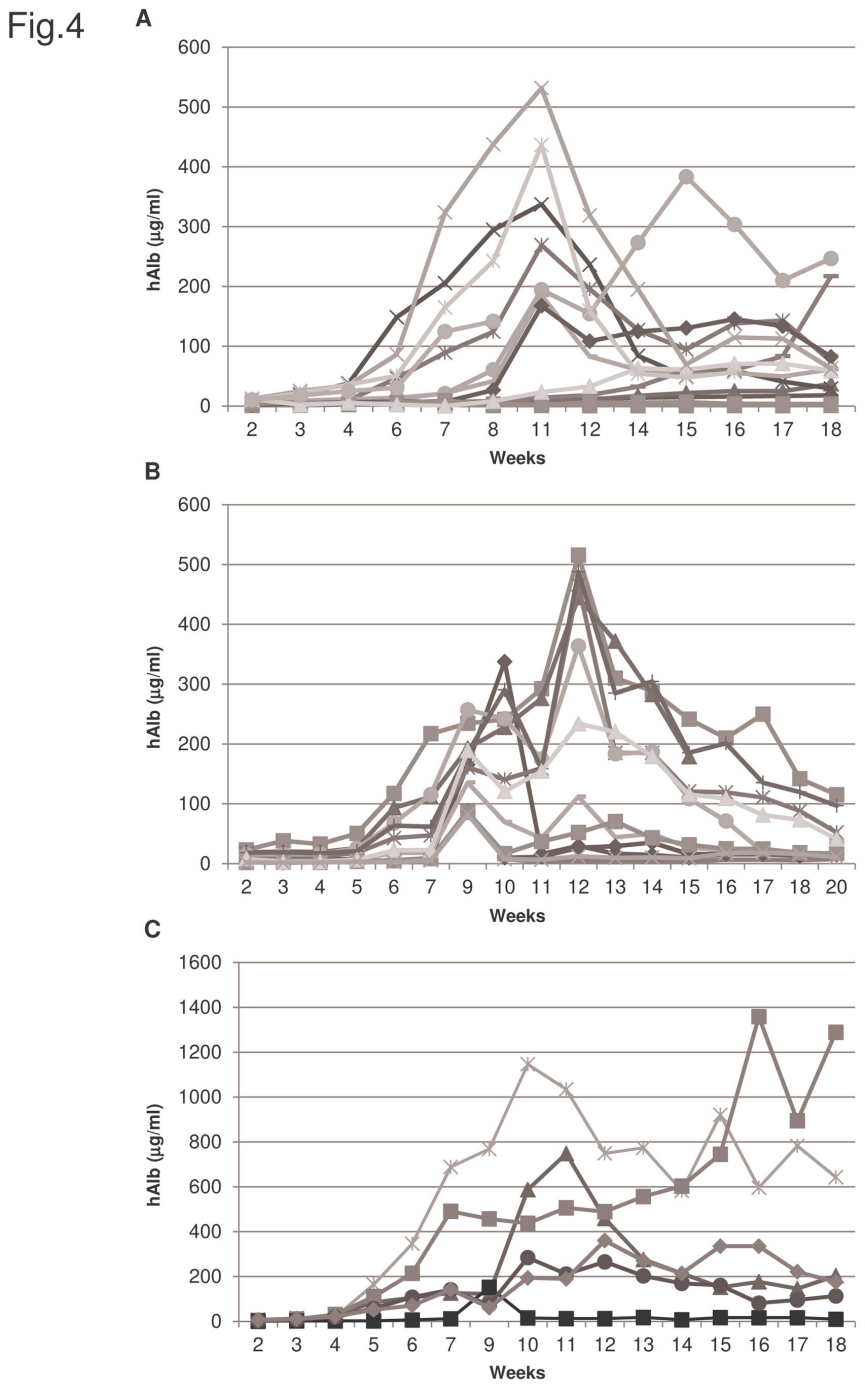
Determination of human albumin concentration in human hepatocyte transplanted mice sera. Peripheral mouse blood was collected once per week after human hepatocyte transplantation and human albumin present in sera was quantified by specific human albumin ELISA analysis. A, B, and C indicate human albumin concentration in three independent experiments with hepatocytes obtained from three different donors. Hepatocytes used in A and C were cryopreserved and plated hepatocytes were used in experiment B. Each line represents human albumin concentration in one animal throughout the experimental period. These three assays are representative of 9 transplantation experiments performed with cryopreserved (4) and plated (5) hepatocytes.

We next evaluated the potential of this model of hepatocellular damage to support the growth of human hepatocytes and to generate humanized livers in immunodeficient mice. We measured the secretion of human albumin to plasma as readout of transplanted primary human hepatocytes engraftment and repopulation. In order to allow for the clearance of the potentially injected human proteins at the time of transplantation we initiate the determinations of human albumin in serum after week 2. We found that serum levels of human albumin increased in many of the transplanted mice from week 3 or 4 after transplantation, observing a heterogeneous engraftment in mice transplanted with the same set of hepatocytes. This increase was sustained for 8-9 weeks to decline gradually thereafter (Figure 4, Figure S4A in File S1). The results show a great heterogeneity in the obtained repopulation, existing animals with very low amount of human albumin detected in the serum to animals that reached levels above 100 µg/ml of human albumin, and in few cases the values were above 1 mg/ml (Figure 4C).

**Figure 5:**
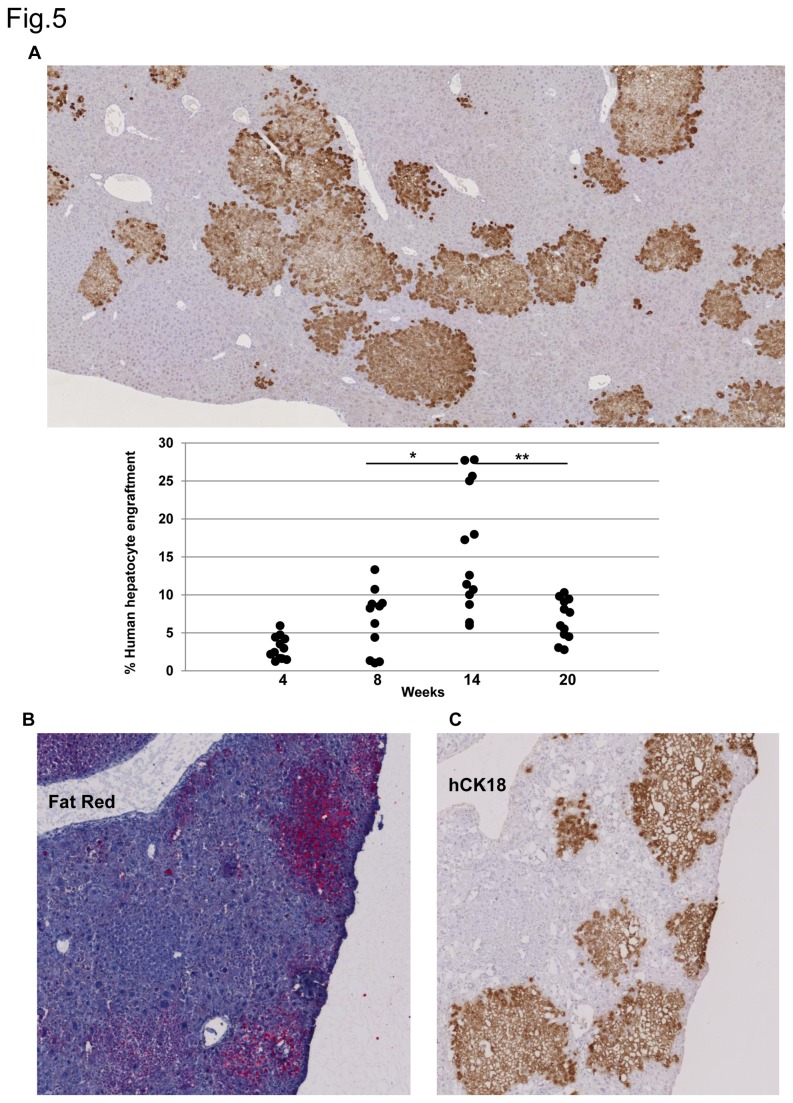
Repopulation of AdTk/GCV treated mice livers with human hepatocytes and analysis of lipid accumulation. A) Mice infected with AdTk and treated with GCV were transplanted with human hepatocytes, animals sacrificed at diverse time points after transplantation and liver sections were immunostained with hCK18 antibody. Liver samples were obtained from 9 different independent experiments and at least two stained liver sections from 6 mice belonging to three independent studies were analyzed at each time point. Obtained images were examined, quantified and represented as the percentage of hepatic section replaced by human hepatocytes (** *p*< 0.01, *** *p*< 0.001, two-tailed Student’s *t*-test). Presented images show human mouse chimeric livers collected at week 14 after transplantation. B) Frozen section of a chimeric liver containing human hepatocytes stained with fat red for detection of lipids (pink colour). C) A serial section of the same liver sample immunostained for hCK18 identifying human hepatocytes. Original magnification x20.

We found that from 5% up to 25% of the murine liver parenchyma could be repopulated with human hepatocytes, with an average repopulation index (RI) of 15% at 14 weeks after transplantation, that declines to 6% six weeks later (Figure 5A). Therefore there is not a perfect correlation between the amount of human albumin detected in mice sera as it is observed when in individually analyzed mice (Figure S4B in File S1) and the percentage liver parenchyma repopulated by human hepatocytes, as the maximum values are reached at week 8 and 14 respectively (Figure 5A, Figure S4A in File S1). But in both cases there is a sustained decline thereafter. Similar RIs were obtained with cryopreserved human hepatocytes and with cells that have been maintained in culture for 5 to 10 days. Taken together, these data indicate that human hepatocytes engraft into mice livers after AdTk/GCV treatment, secrete human serum albumin and proliferate to form regenerating nodules.

To study the structure of the chimeric livers, we collected the organs at 14-18 weeks for histological analysis. We found that some hepatocyte nodules were clearly distinguished from the surrounding parenchyma by fainter staining and vacuolarized appearance of some of the hepatocytes (Figure S5 in File S1). Immunohistochemical staining for human albumin (hAlb) and human cytokeratin 18 (hCk18) verified that these nodules were composed by human hepatocytes. In addition, Fat Red staining showed increased lipid droplets in many hCK-18-positive human hepatocyte nodules being the steatosis more intense in the big nodules (Figure 5B). These cytological changes were similar to those described by other authors in human hepatocytes transplanted to the murine liver [22,23].

**Figure 6:**
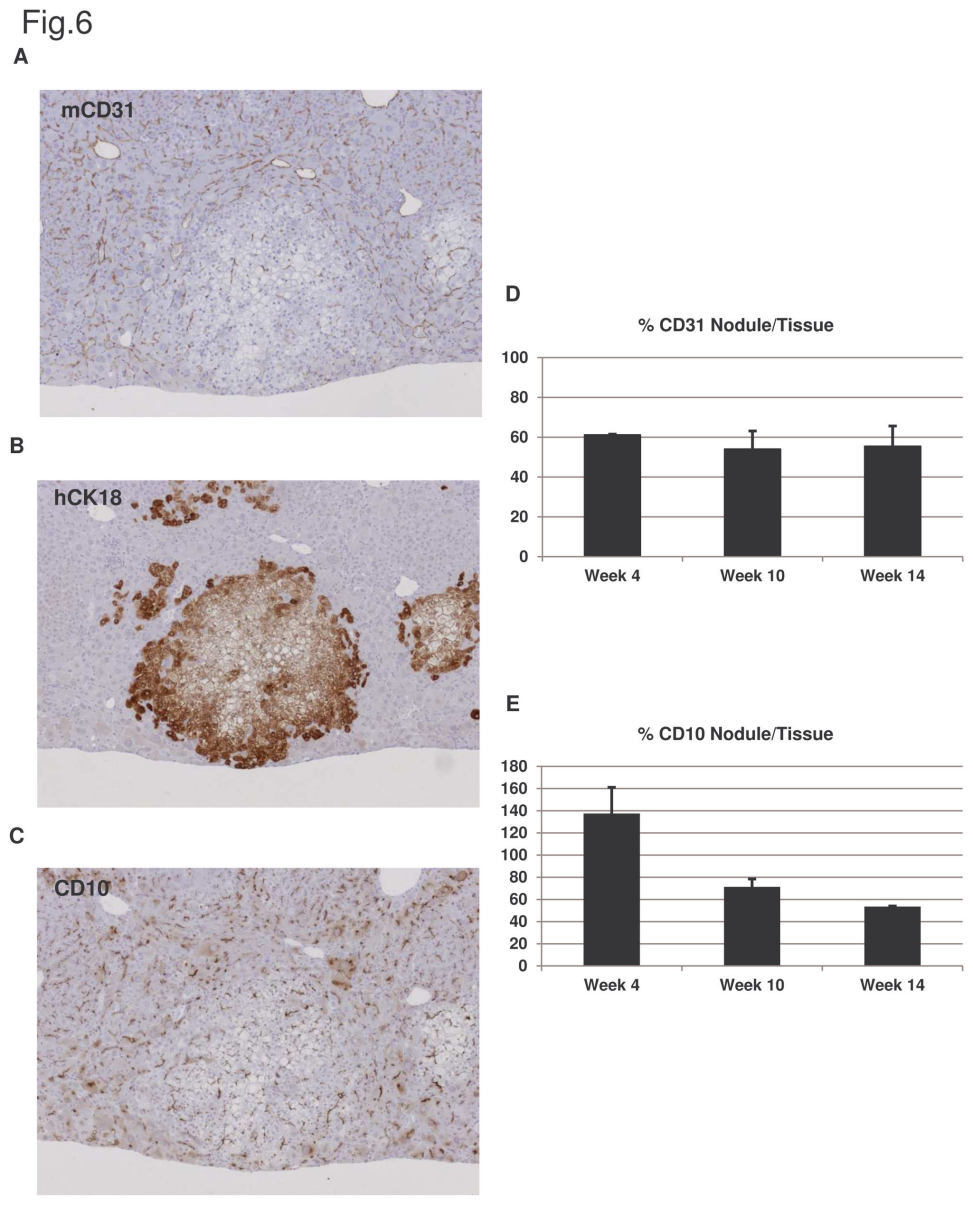
Comparative analysis of endothelial cells and bile canaliculi present in human nodules and host parenchyma. Serial liver sections of mice transplanted with human hepatocytes for 4, 10 and 14 weeks were analyzed for the presence of endothelial cells (mCD31) (A), human CK18 (B) and bile canaliculi (CD10) (C) by immunohistochemistry. Quantification of mouse endothelial cells (D) and bile canaliculi (E) present in human regenerative nodules respect to murine host parenchyma at different time points post-engraftment were performed. Two hepatic sections from three animals were analyzed at each time point. Original magnification x100.

We also observed that, unlike the regenerating nodules of transplanted GFP-positive murine hepatocyte, the nodules formed by transplanted human hepatocytes showed reduced number of endothelial cells (CD31+ cells) (Figure 6A, 6D). And the paucity of CD31+ cells persisted from week 4 until the end of the study period (Figure 6D). Moreover, this effect is independent of the state of the human hepatocytes as 4 weeks after transplantation engrafted human hepatocytes are healthy and indistinguishable from the host parenchyma (Figure S6 in File S1). Because of this defect in the parenchyma/endothelial balance, we next evaluated the polarization of the human hepatocytes by assessing the localization of CD10, a protein that corresponds to a cell membrane metallopeptidase normally present at the apical pole of hepatocytes being inserted into the canalicular membrane [30]. We found that the transplanted human hepatocytes were well polarized at week 4 after transplantation but the amount of CD10 detected in the nodules decreased over time (Figure 6E).

Thus our data show that, in the long run, the human hepatocytes transplanted to the murine livers, experience metabolic alterations, do not polarize properly and suffer from defective angiogenesis.

### HBV and HCV infection

**Figure 7:**
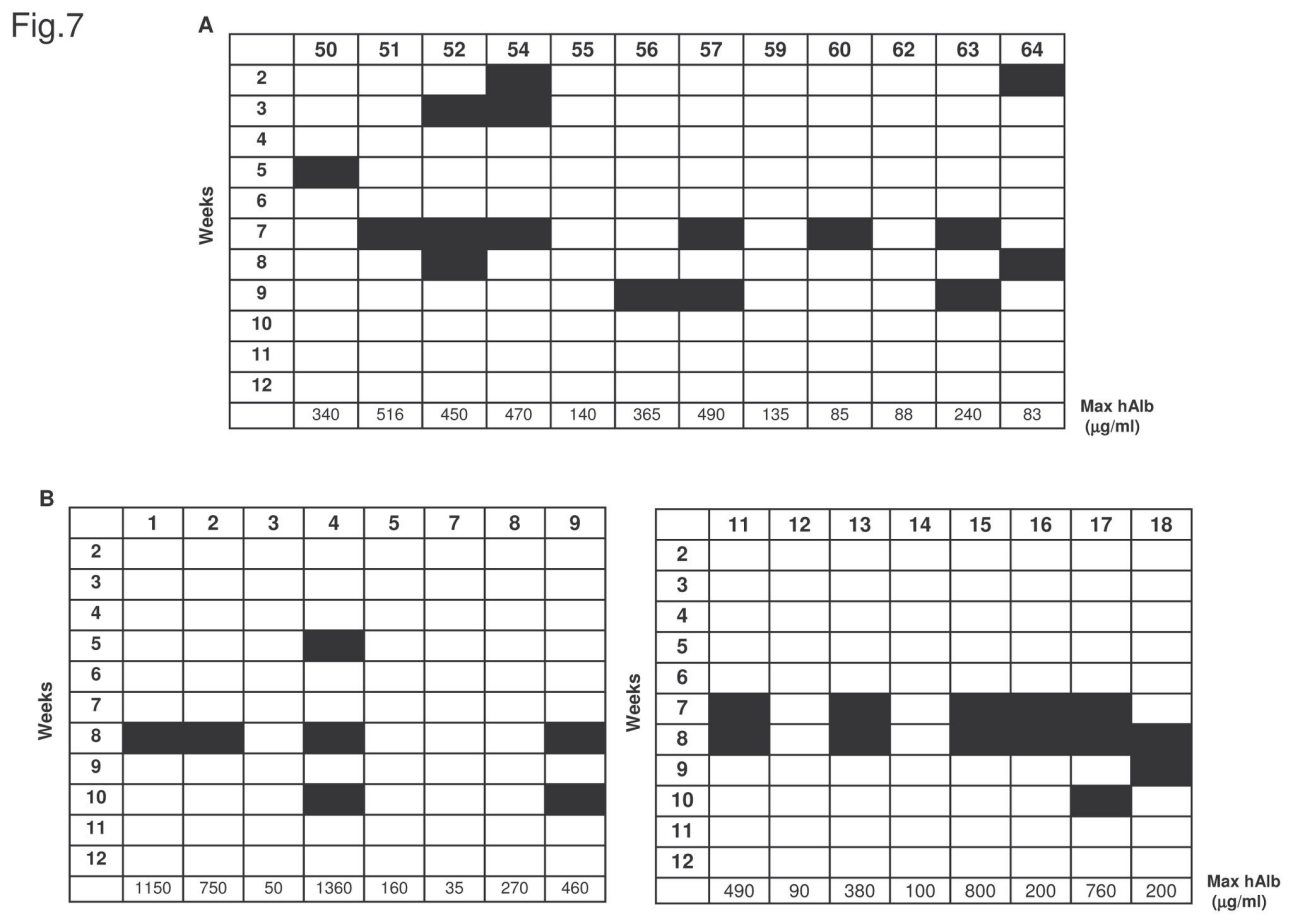
HBV and HCV infection of mice transplanted with human hepatocytes. Mice transplanted with human hepatocytes for 8 weeks were inoculated with HBV (A) or HCV (B) human chronic carriers sera, animals were bled weekly and analyzed by PCR for the presence of HBV or HCV viral particles. In the table are indicated in the first line animal identification, with black boxes when viral genomes were detected in mice sera and the maximum human albumin concentration detected in each animal along the experiment. Viral presence was determined when the PCR reaction gave a positive result without quantifying it (Figure S7 in File S1). In B, there are presented two independent HCV inoculation experiments.

Chimeric mice with human hepatocyte engraftments are the only small animal models that can be robustly infected with HBV and HCV. These animals are differentially susceptible to HBV and HCV infection, requiring smaller amounts of human hepatocyte engraftment (serum human albumin levels > 0.3 mg/ml) for a successful HBV infection compared to HCV (serum human albumin levels > 1 mg/ml) [31]. Despite achieving a reduced liver repopulation index than what has been previously described as required for efficient HCV and HBV infection, we evaluated the susceptibility of the AdTk/GCV-treated mice, transplanting them with human hepatocytes to be infected by HBV and HCV. To this end, we inoculated chimeric mice with HBV- or HCV-infected patient sera at 7-8 weeks after the transplantation of human hepatocytes. Once per week, we measured the serum viral presence to measure viral production in several infected mice at different time points after viral inoculation (Figure 7 and Figure S7 in File S1). We found that the viral production could be detected sporadically in some of the inoculated animals. Consistent with previous reports, we found a positive correlation between the viral production and the rate of albumin production [12,31] (Figure 7). In conclusion, the AdTk/GCV-based chimeric mouse model cannot support a robust HCV and HBV infection.

## Discussion

The use of mice with chimeric humanized livers has been demonstrated to be a useful approach for the study of human liver diseases. The generation of these chimeras has been hampered by the complexity of using genetically modified animals that are cross-bread with severe immunodeficient mice to give rise to models of chronic liver damage in a background of immune permisitivity for xenogenic grafts [11]. In this study, we have generated chimeric mice in a much easier way, simply causing liver damage with AdTk inoculation followed by GCV administration to immunodeficient mice. Interestingly we observed that this treatment induced persistent hepatocellular injury lasting for several weeks, as has been previously described in Tk transgenic animals [27,28], allowing for liver repopulation with allogenic or xenogenic hepatocytes. Liver repopulation with transplanted hepatocytes following AdTk/GCV administration is facilitated by the fact that this regime impairs the regenerative capacity of the native mature hepatocytes. This notion is supported by the proliferation in the recipient liver of oval cells, a progenitor cell compartment which is activated when normal hepatocytes fail to regenerate [32].

In our model, transplanted murine hepatocytes were able to replace between 20% to 50% of the hepatic parenchyma and human hepatocytes up to 15%. It seems possible that the limited organ replacement by the transplanted hepatocytes is due to the transient nature of liver injury, allowing competition of autochthonous liver cells which start proliferating by week 10 when the intensity of the Tk/GCV-induced liver damage starts to subside. Accordingly, there is a sustained decrease in human albumin detected in mice sera 10-12 weeks after human hepatocyte transplantation that is accompanied by a reduction in the RI 4-6 weeks later. This is different from what occurs in models employing genetically modified mice where hepatocellular damage is sustained over the time allowing for transplanted cells to substitute near 90% of the total parenchyma [6,10,12,13,31,33]. Due to these limitations, the partial humanization of the murine liver obtained with our system allowed only a transient HBV and HCV infection of the chimeric mice, blocking its use in experiments that require a massive repopulation of the liver as human hepatotropic infections and toxicological studies. Thus, it would be necessary to obtain a more prolonged mouse liver injury in order to allow a higher human hepatocyte repopulation. This could be achieved making use of a viral vector with a sustained Tk expression that would allow a GCV readministration 6-8 weeks after liver injury induction, once host liver is recovering. However this approach could be limited by the bystander effect associated to the Tk-GCV treatment [34].

Another reason for limitations in liver replacement by transplanted human hepatocytes is the inability of these cells to interact appropriately with adjacent cells of the liver parenchyma. Typically the nodules formed by regenerating human hepatocytes in the murine liver were characterized by reduced vascularization and paucity of CD31 positive cells inside the nodules, possibly as result of impaired intercellular crosstalk in the chimeric liver microenvironment [24]. This is in clear contrast with the normal presence of CD31+ cells within regenerative nodules when the transplanted cells were murine hepatocytes. The defective vascularization observed in the nodules of transplanted human hepatocytes is associated with signs of metabolic stress in the transplanted cells including steatosis and cell vacuolarization that may explain the RI decrease from week 14. This effect is more apparent in the center than in the periphery of human regenerative nodules, suggesting the absence of a substantial Tk-GCV bystander effect promoting this damage. Nevertheless we cannot exclude the possibility of a subtle effect of GCV on human hepatocytes that could damage them.

Moreover the human liver cells lose their polarity showing abnormal distribution of the canalicular protein CD10 [30] and eventually a marked decrease of its expression. These alterations in the architecture of the regenerating nodules of human hepatocytes might account for the fact that these humanized livers can support HBV and HCV infections for a limited period of time as hepatocyte polarization is required for efficient viral infection and production of viral particles [35,36]. Therefore this animal model can help to decipher the mechanisms that govern endothelial cell proliferation and recruitment to the regenerative nodules when transplanted human hepatocytes are proliferating and how this affects the generation of a correct hepatic architecture. Furthermore, expression of human proteins [23] or transplantation of human hepatocytes with human sinusoidal or endothelial cells can promote a proper humanized regenerative nodule assembly. Consequently the obtained humanized murine livers could predict more accurately the responses in human livers.

Thus we have described, for the first time, the efficient engraftment of human hepatocytes into murine livers using a model of drug-induced liver damage not requiring genetically modified animals for cross-breeding with immunodeficient mice. This method significantly simplifies the procedure of generating humanized livers for use in pharmacological and human hepatocyte studies and it opens the possibility of generating chimeric livers in animals not modified genetically, simplifying and reducing time and expenses required for generation of mice with humanized livers. However, additional modifications of the system are needed, including co-transplantation with progenitor endothelial cells and/or human macrophages or other cell types, in order to achieve more abundant vascularization of the regenerative nodules, improved hepatocyte polarization and more efficient humanization of the murine liver.

## Supporting Information

File S1 Supporting figures.
**Figure S1**. Hepatic identification of leukocyte, macrophages and neutrophils infiltration after AdTk/GCV mediated liver injury. The images represent liver sections of control mice, mice infected with AdTk alone (AdTk) or treated with GCV (AdTk + GCV). Images of mice treated with GCV alone are similar to the observed in control and AdTk mice. They show localization of leukocytes (CD45 antigen), macrophages and neutrophils in mouse parenchyma 4 weeks after GCV administration. Original magnification x20. Quantification of Kupffer cells and neutrophils were performed in three stained liver sections from 5 animals/time point/group using FIJI imaging software and obtained results were normalized with control group values. X axis represents the days post GCV administration and liver damage induction while Y axis represents normalized ratio between positive area and total area in arbitrary units. All AdTk+GCV groups present statistically significant differences compared to the Control and AdTk groups (*** *p*< 0.001, two-tailed Student’s *t*-test).
**Figure S2**. Analysis of oval cells, activated stellate cells and collagen deposition after AdTk/GCV mediated liver injury. Represent liver sections of mice without any treatment (Control), mice infected with recombinant adenovirus expressing HSV-Tk (AdTk) alone or infected with AdTk and treated with GCV (AdTk + GCV). Images obtained from mice treated with GCV alone livers are similar to the observed in control and AdTk mice. Murine oval cells were stained with A6 antibody and activated stellate cells with α-smooth muscle actin antibody 4 weeks after GCV administration (original magnification x40). Collagen deposition was demonstrated using picrosirius red staining. Original magnification x20.
**Figure S3**. Presence of endothelial cells and bile canaliculi in GFP mouse hepatocyte nodules. Hepatic serial sections from a mouse transplanted for 14 weeks with murine GFP expressing hepatocytes were analyzed for (A) endothelial cells (mCD31) and (C) bile canaliculi (CD10). Hepatocytes derived from transplanted hepatocytes are identified as GFP positive cells (B). Original magnification x100.
**Figure S4**. Quantification of human albumin concentration in human hepatocyte transplanted mice and analyzed for RI. (A) Peripheral blood was collected when animals were sacrificed and human albumin present in sera was quantified by specific human albumin ELISA analysis. The analyzed animals are the same used in the RI study (* *p*< 0.05, two-tailed Student’s *t*-test). (B) Measurement of human albumin concentration in mice sera and human hepatocyte engraftment in individual mice.
**Figure S5**. Identification of humanized regenerative nodules in the liver of mice transplanted with human hepatocytes. Serial liver sections from a mouse transplanted with human hepatocytes for 16 weeks after AdTk/GCV treatment were stained for hCK18 (A), HE (B) and hAlb (C) detection. Original magnification x100.
**Figure S6**. Presence of endothelial cells and bile canaliculi in human hepatocyte nodules 4 weeks after transplantation. Hepatic serial sections from two mice transplanted for 4 weeks with human hepatocytes were haematoxylin-eosyn stained and analyzed for human hepatocytes (hCK18), endothelial cells (mCD31) and bile canaliculi (CD10). (A) And (B) represent hepatic sections from two different mice. Original magnification x200.
**Figure S7**. HBV and HCV infection of mice transplanted with human hepatocytes. Mice transplanted with human hepatocytes for 8 weeks were inoculated with HBV human chronic carriers sera, animals were bled weekly and analyzed by PCR for the presence of HBV (A) and HCV (B) viral particles. In A, there are presented PCR results at week 5, 6, 7 and 9 after HBV inoculation and in B, there are presented PCR results at week 6, 7, 8, 9 and 10 after HCV inoculation. The numbers indicate animal identification, W is PCR reaction from non-infected animal sera and C+ is PCR positive control from patient sera used for inoculation.
(PDF)
