# Supplementary figures and images for: Usage of Adenovirus Expressing Thymidine Kinase Mediated Hepatocellular Damage for Enabling Mouse Liver Repopulation with Allogenic or Xenogenic Hepatocytes
Source: PLoS One. 2013 Sep 24;8(9):e74948. doi: 10.1371/journal.pone.0074948 (PMC3782477; doi:10.1371/journal.pone.0074948)

Fig.S1

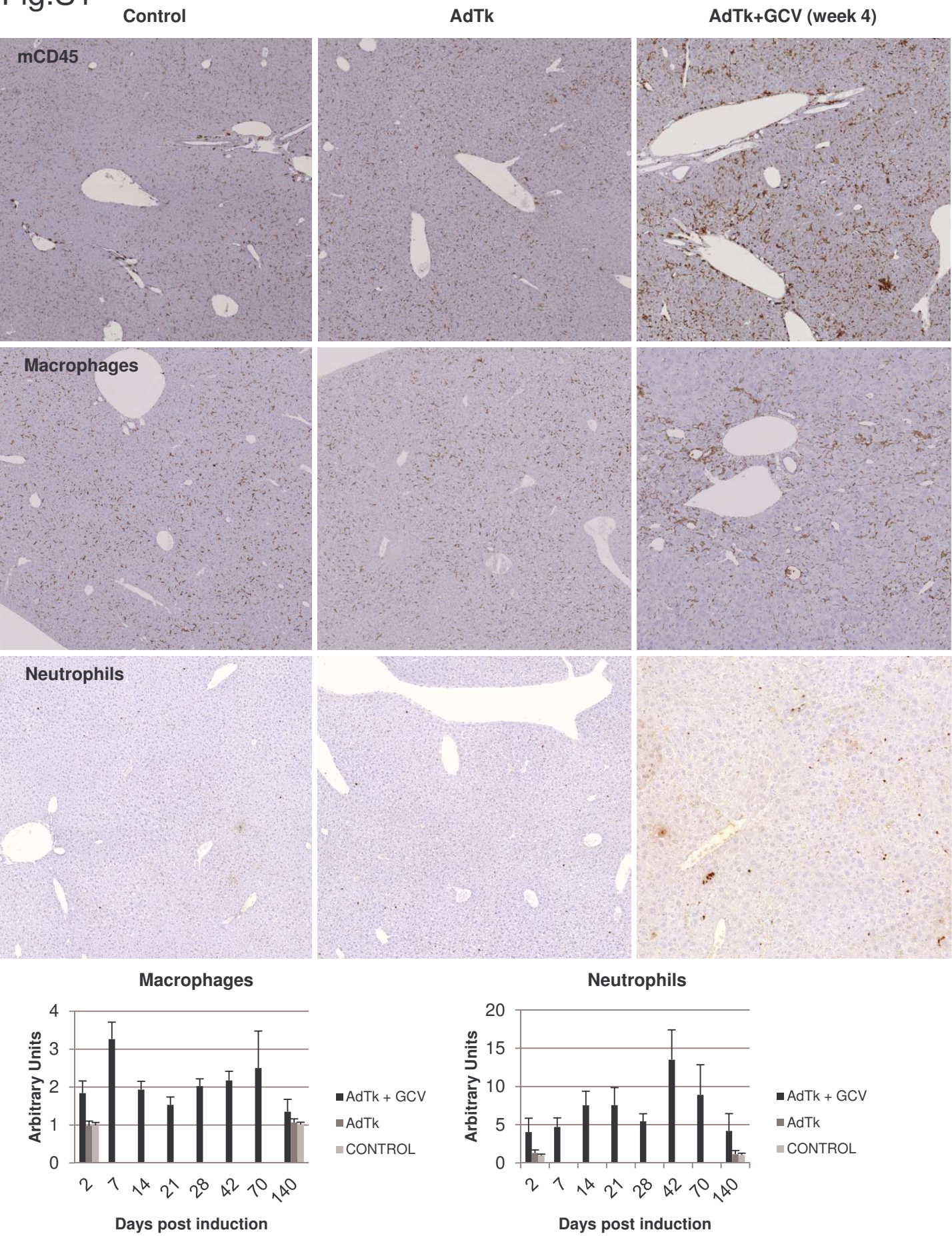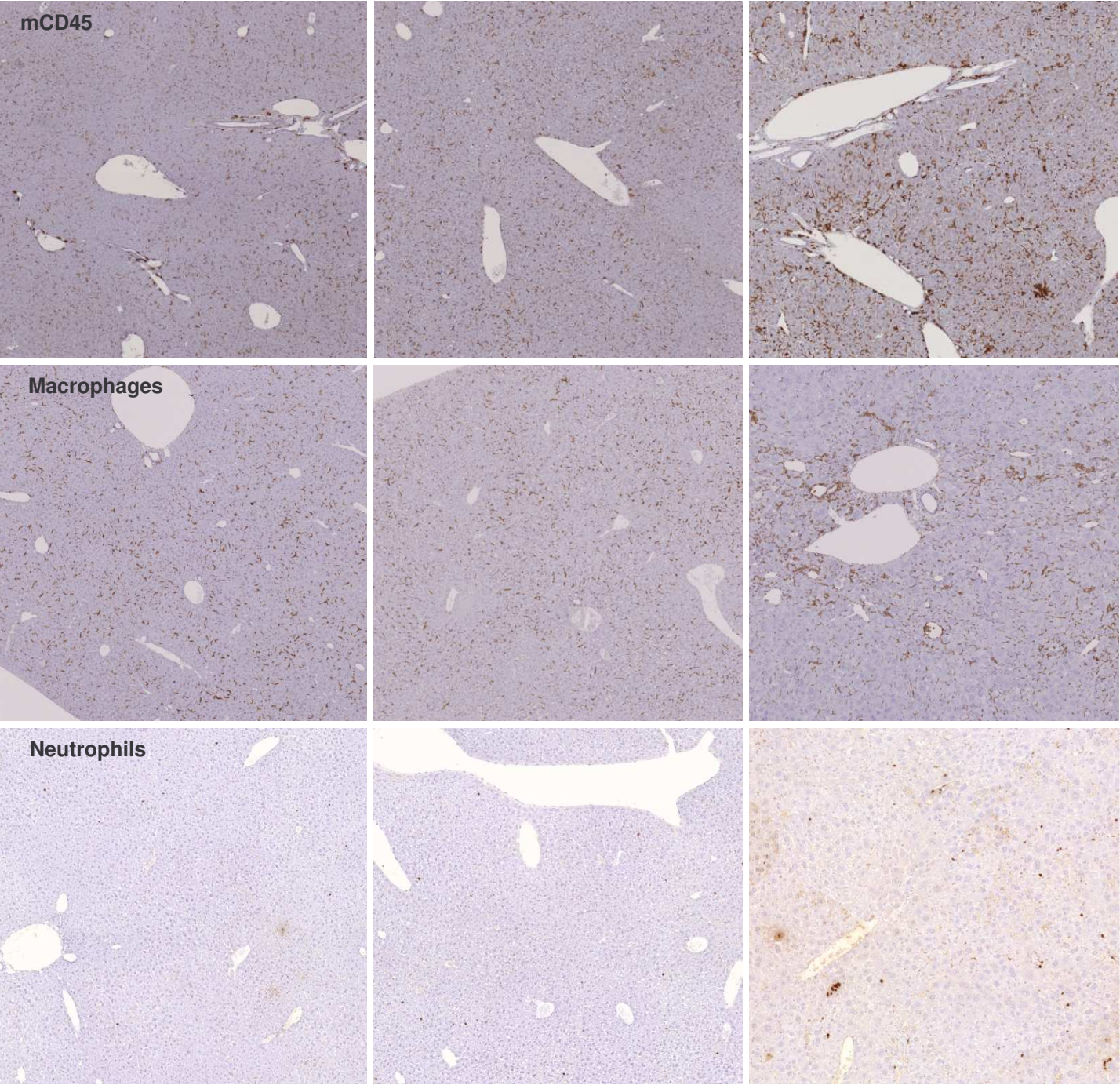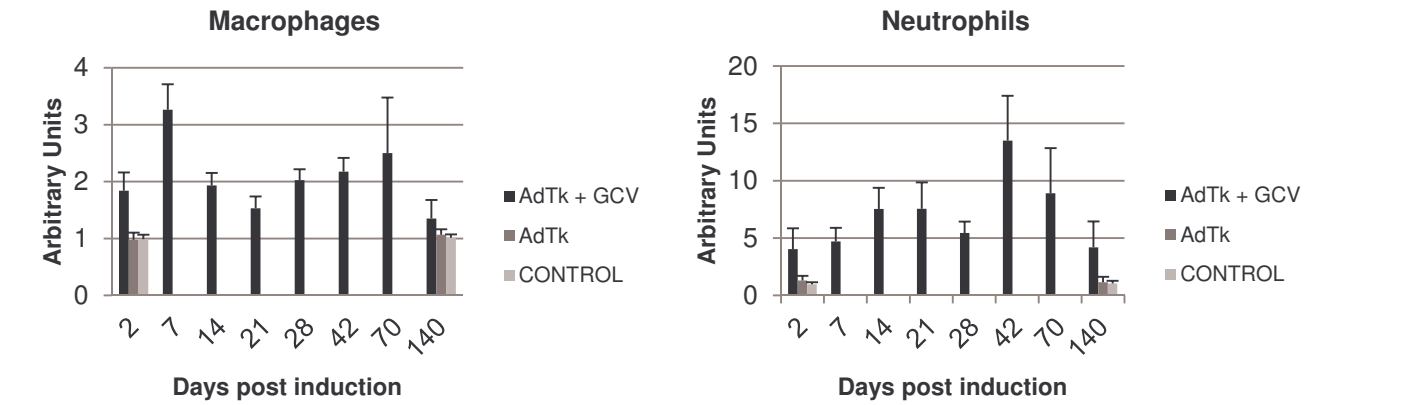

Fig.S2

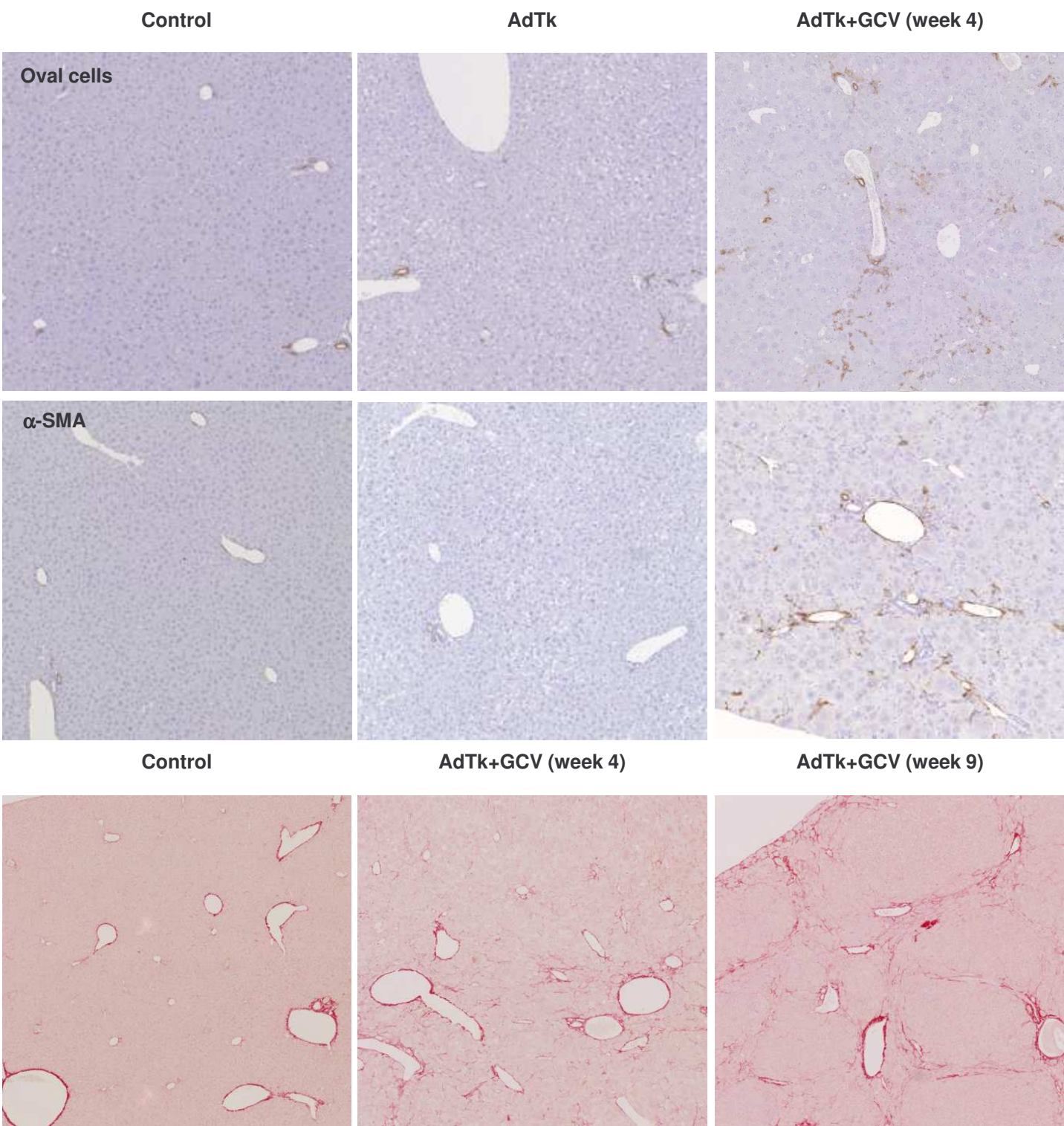

Fig.S3

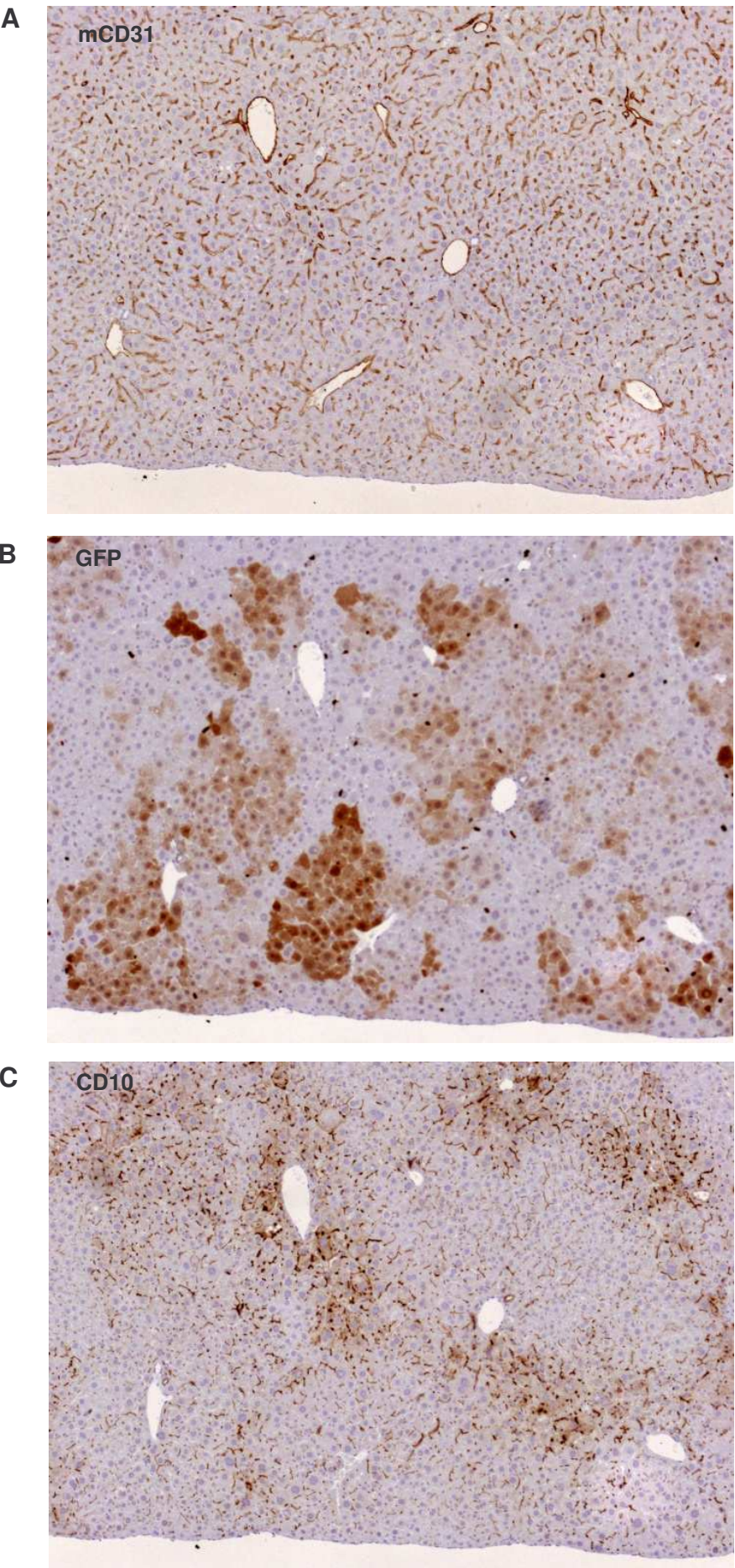

Fig.S4

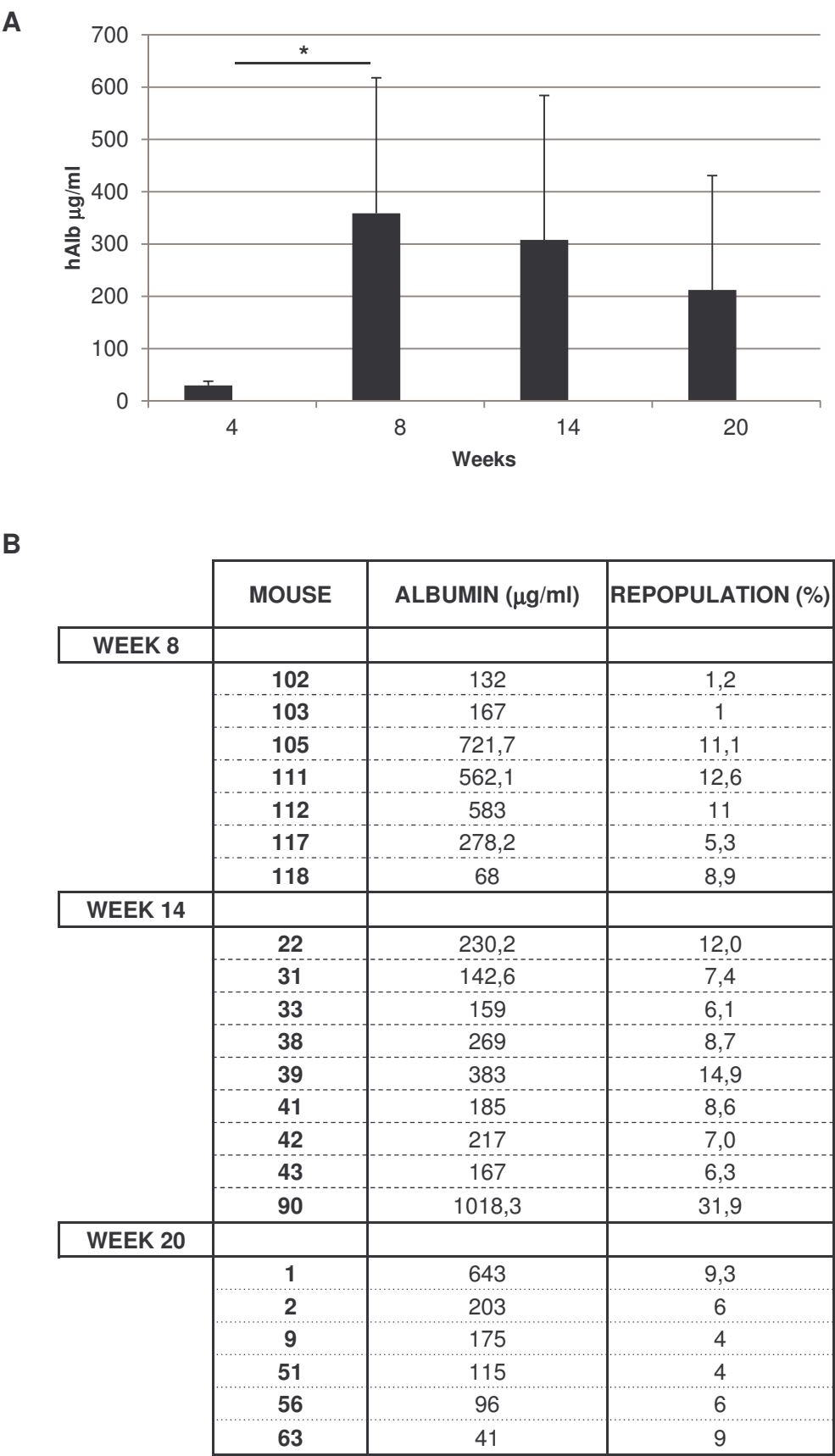

Fig.S5

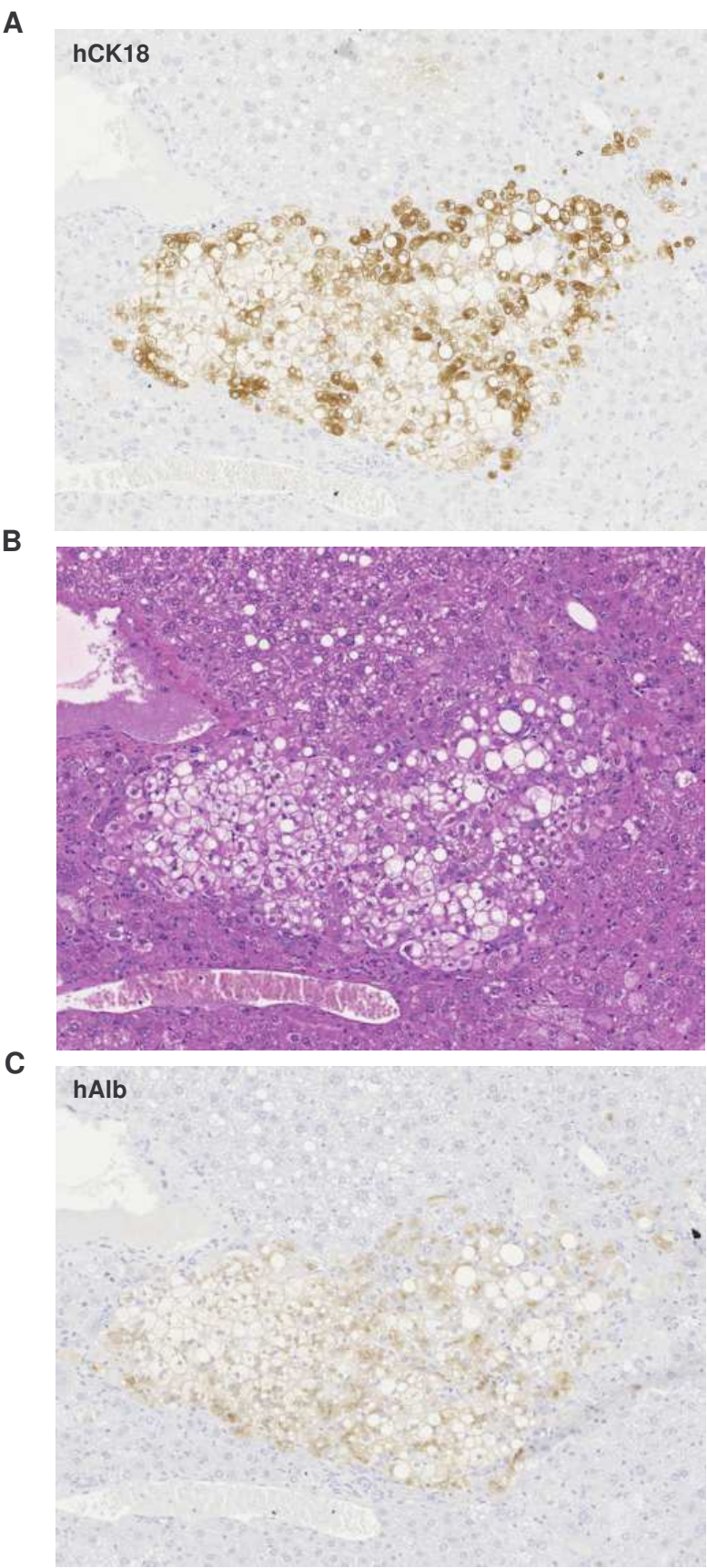

Fig.S6

A

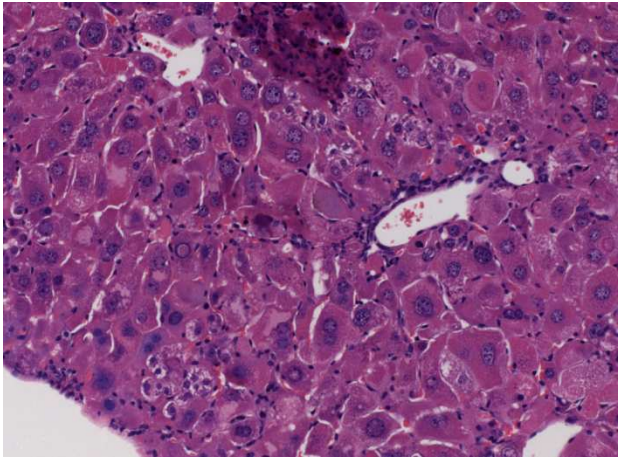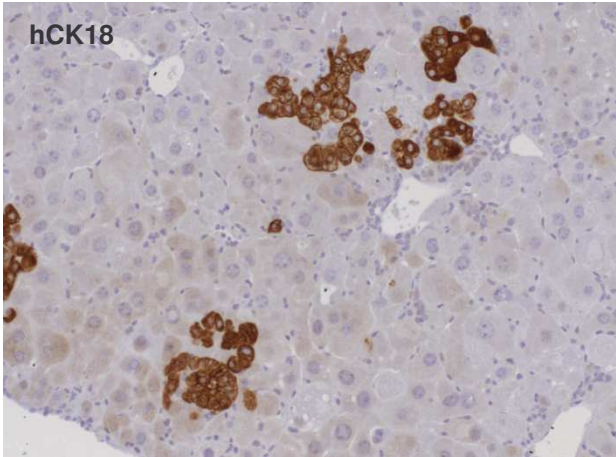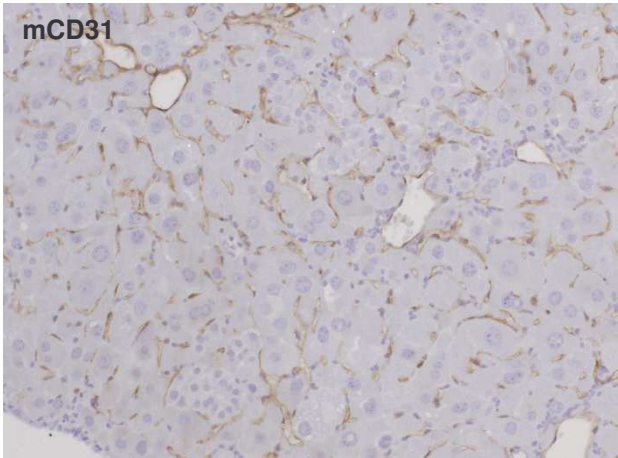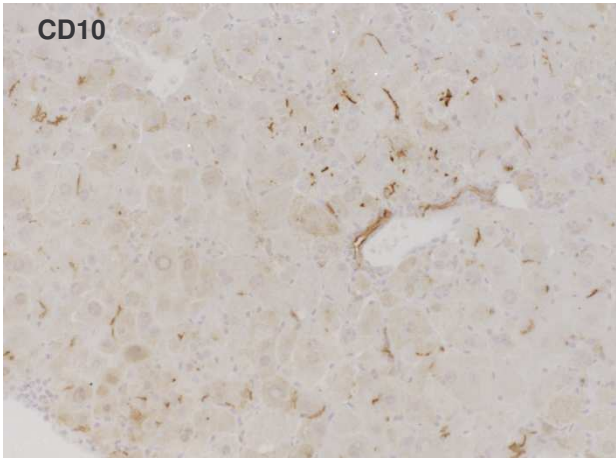

B

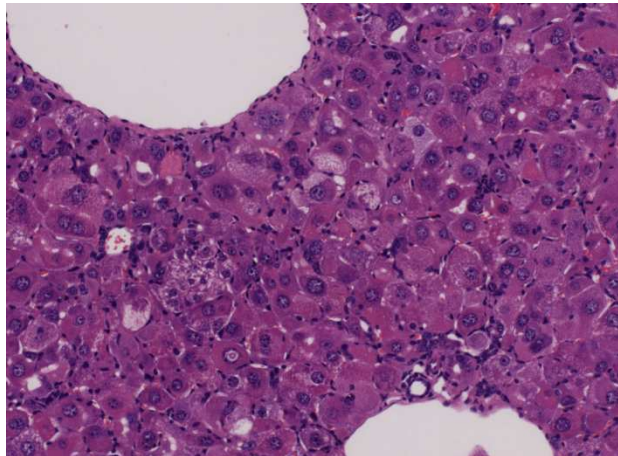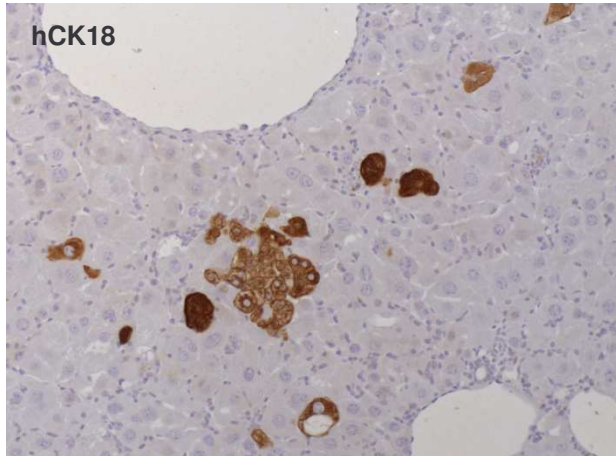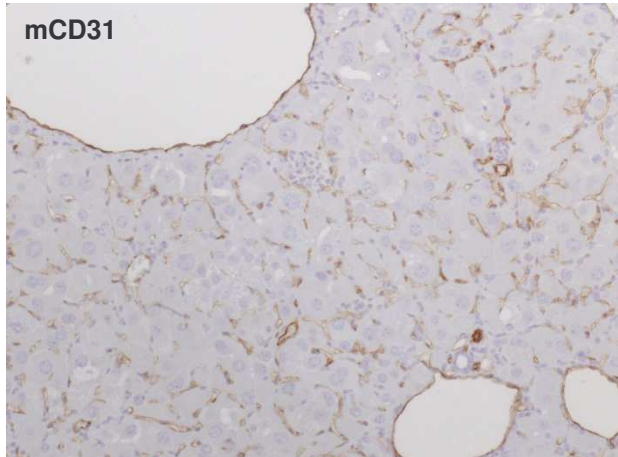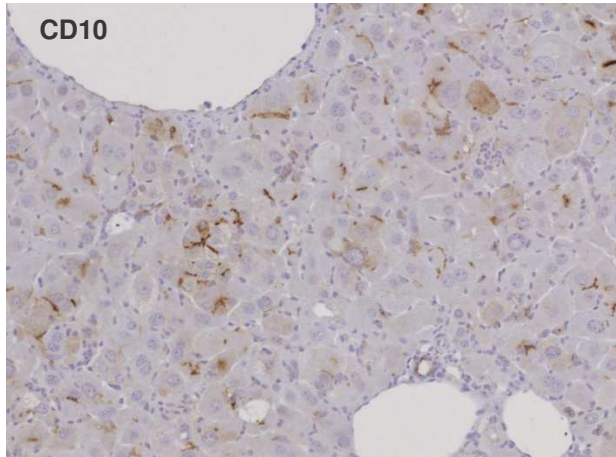

Fig.S7

A

Week 5

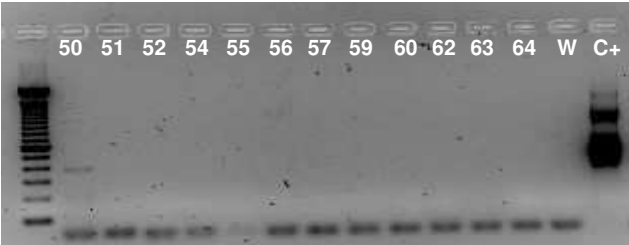

Week 6

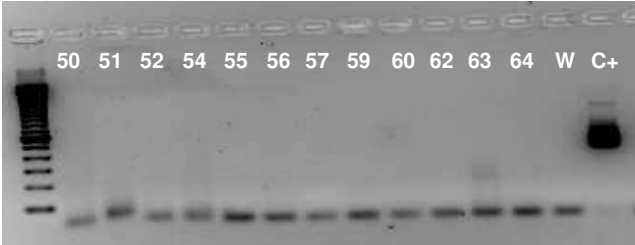

Week 7

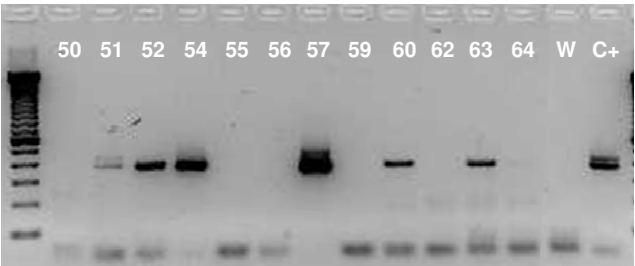

Week 9

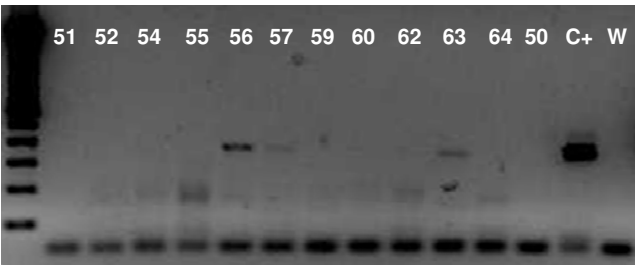

B

Week 6

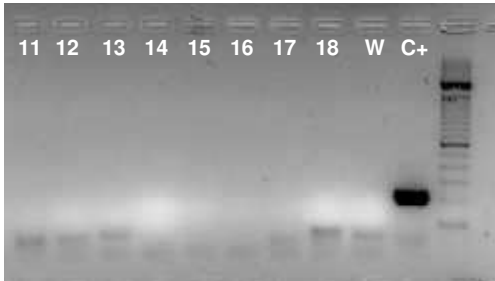

Week 7

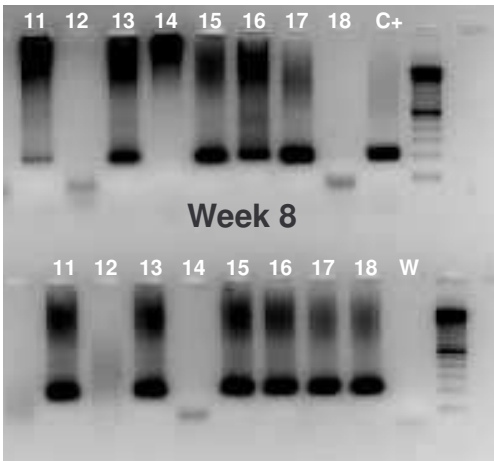

Week 8

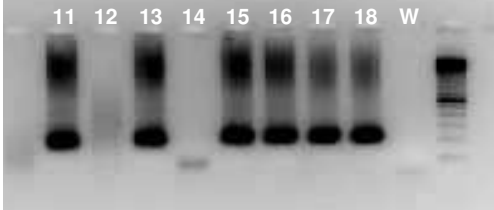

Week 9

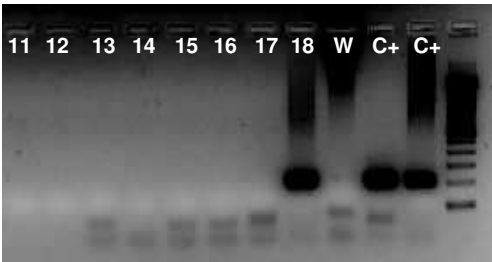

Week 10

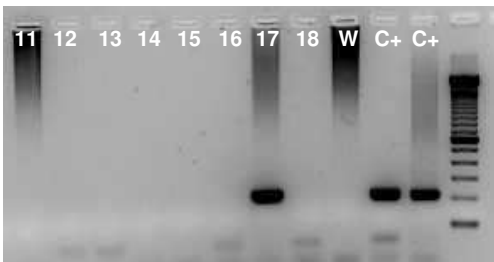

Supplement: File S1 — Supporting figures. Figure S1. Hepatic identification of leukocyte, macrophages and neutrophils infiltration after AdTk/GCV mediated liver injury. The images represent liver sections of control mice, mice infected with AdTk alone (AdTk) or treated with GCV (AdTk + GCV). Images of mice treated with GCV alone are similar to the observed in control and AdTk mice. They show localization of leukocytes (CD45 antigen), macrophages and neutrophils in mouse parenchyma 4 weeks after GCV administration. Original magnification x20. Quantification of Kupffer cells and neutrophils were performed in three stained liver sections from 5 animals/time point/group using FIJI imaging software and obtained results were normalized with control group values. X axis represents the days post GCV administration and liver damage induction while Y axis represents normalized ratio between positive area and total area in arbitrary units. All AdTk+GCV groups present statistically significant differences compared to the Control and AdTk groups (*** p< 0.001, two-tailed Student’s t-test). Figure S2. Analysis of oval cells, activated stellate cells and collagen deposition after AdTk/GCV mediated liver injury. Represent liver sections of mice without any treatment (Control), mice infected with recombinant adenovirus expressing HSV-Tk (AdTk) alone or infected with AdTk and treated with GCV (AdTk + GCV). Images obtained from mice treated with GCV alone livers are similar to the observed in control and AdTk mice. Murine oval cells were stained with A6 antibody and activated stellate cells with α-smooth muscle actin antibody 4 weeks after GCV administration (original magnification x40). Collagen deposition was demonstrated using picrosirius red staining. Original magnification x20. Figure S3. Presence of endothelial cells and bile canaliculi in GFP mouse hepatocyte nodules. Hepatic serial sections from a mouse transplanted for 14 weeks with murine GFP expressing hepatocytes were analyzed for (A) endotheli [file pone.0074948.s001.pdf]
